# Modulation of the Microtubule Network for Optimization of Nanoparticle Dynamics for the Advancement of Cancer Nanomedicine

**DOI:** 10.3390/bioengineering7020056

**Published:** 2020-06-14

**Authors:** Aaron Bannister, Dushanthi Dissanayake, Antonia Kowalewski, Leah Cicon, Kyle Bromma, Devika B. Chithrani

**Affiliations:** 1 Department of Physics and Astronomy, University of Victoria, Victoria, BC V8P 5C2, Canada; aaronhenrybannister@gmail.com (A.B.); dushanthidissanayake@uvic.ca (D.D.); lcicon@uvic.ca (L.C.); kbromma@uvic.ca (K.B.); 2 Department of Physics, Simon Fraser University, Burnaby, BC V5A 1S6, Canada; antonia_kowalewski@sfu.ca; 3 Centre for Advanced Materials and Related Technologies (CAMTEC), University of Victoria, Victoria, BC V8P 5C2, Canada; 4 Centre for Biomedical Research, University of Victoria, Victoria, BC V8P 5C2, Canada

**Keywords:** nanoparticles, docetaxel, microtubules, nanomedicine, cancer

## Abstract

Nanoparticles (NPs) have shown promise in both radiotherapy and chemotherapy. NPs are mainly transported along cellular microtubules (MTs). Docetaxel (DTX) is a commonly used chemotherapeutic drug that can manipulate the cellular MT network to maximize its clinical benefit. However, the effect of DTX on NP behaviour has not yet been fully elucidated. We used gold NPs of diameters 15 and 50 nm at a concentration of 0.2 nM to investigate the size dependence of NP behaviour. Meanwhile, DTX concentrations of 0, 10 and 50 nM were used to uphold clinical relevance. Our study reveals that a concentration of 50 nM DTX increased NP uptake by ~50% and their retention by ~90% compared to cells treated with 0 and 10 nM DTX. Smaller NPs had a 20-fold higher uptake in cells treated with 50 nM DTX vs. 0 and 10 nM DTX. With the treatment of 50 nm DTX, the cells became more spherical in shape, and NPs were redistributed closer to the nucleus. A significant increase in NP uptake and retention along with their intracellular distribution closer to the nucleus with 50 nM DTX could be exploited to target a higher dose to the most important target, the nucleus in both radiotherapy and chemotherapy.

## 1. Introduction

The use of nanoscale materials in cancer treatment to overcome challenges in current therapeutic options led to the interdisciplinary field of cancer nanomedicine [1]. Radiotherapy and chemotherapy are the most employed therapeutic strategies, in addition to surgery, in the treatment of cancer. The main issue with both chemotherapy and radiotherapy is the normal tissue toxicity. For example, only a small portion of the drug actually arrives at the tumour site, necessitating the administration of a higher drug dose. Consequently, this often causes toxic, dose-limiting side effects in chemotherapy. The nanoparticle (NP)-based delivery of chemotherapeutic drugs is being explored to improve drug delivery to the tumour site while minimizing the injected dose. The development of over 15 approved nanomedicine drugs highlights the progress made in NP-driven chemotherapy [2,3]. Most of these NP delivery systems are lipid or polymer based. Recently, progress has been made in using inorganic NPs, such as gold nanoparticles (GNPs), for the efficient delivery of anticancer drugs in chemotherapy [4,5,6].

In radiotherapy, NP-driven radiosensitization strategies that target high-Z materials to tumour cells have been pursued to improve the local radiation dose while minimizing the damage to surrounding healthy tissue [7]. The interaction of high-Z materials with therapeutic X-ray photons results in an increase in the local cross-section of cell damaging species, such as free radicals and low energy electrons [8,9]. Inorganic NP systems such as GNPs, silver NPs, gadolinium-based NPs, lanthanide-based NPs, and titanium oxide nanotubes have been reported as radiosensitizers [10,11,12,13,14,15,16]. Gadolinium-based NPs offer an innovative approach because of their capacity to act as a radiosensitizer as well as a powerful contrast agent in magnetic resonance imaging [15]. Furthermore, the high Z-nature of silver-based NPs along with their antimicrobial properties made them a good candidate in radiotherapy [16]. However, GNPs are the most widely used NP system in radiotherapy due to their ease of production, advantageous surface chemistry, and biocompatibility [14,17,18,19].

In order to cause maximal cell damage in any of the NP-based therapeutic application discussed above, further optimization of NPs’ intracellular behaviour (e.g., pertaining to uptake, distribution, and retention) within cancer cells is critical. For example, having a significantly larger number of GNPs closer to the nucleus will enhance the delivery of drugs and radiation dose to the most important target, the nucleus, in chemotherapy and radiotherapy, respectively. Manipulation of NPs’ size and surface properties has been widely used to optimize the uptake of NPs in cancer cells in preclinical applications. Recent studies have exploited the cell cycle to further improve cellular NP uptake [20,21]. For example, arresting the cell cycle in the G2/M phase could result in a higher intracellular accumulation of NPs (see Figures S1–S3) [21]. It is known that taxol-based chemotherapeutic drugs such as docetaxel (DTX) can arrest the cell cycle in the G2/M phase through the stabilization of microtubules (MTs). The stabilization of MTs prevents spindle assembly during the mitosis phase of the cell cycle and thus prevents cell division [22,23]. Most NP systems enter the cell via endocytosis and are transported in vesicles along MTs [24]. However, the change in NP behaviour in the presence of the MT stabilizing agent, DTX, has not yet been fully elucidated. Hence, this study focused on answering fundamental questions regarding the integration of DTX and NPs to achieve improved outcomes in NP-based therapeutics. In the next section, the role of the MT network in the cellular transport of NPs in the absence of the MT stabilizing agent, DTX, will be discussed.

**Figure 1:**
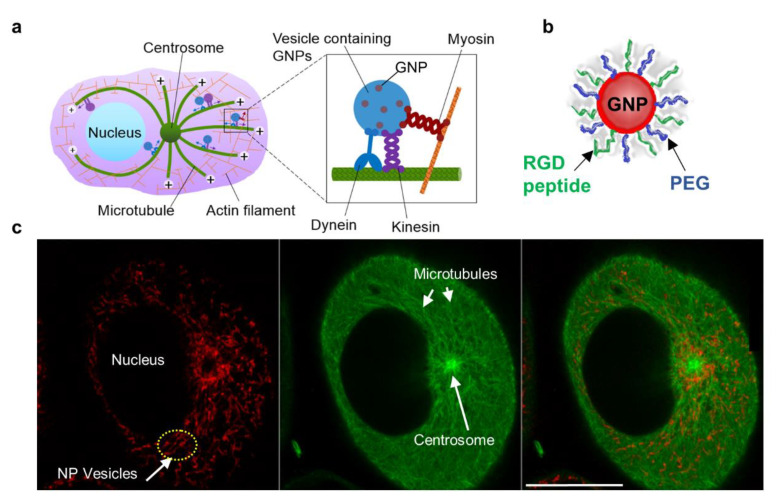
Intracellular transport of NPs. (**a**) Schematic diagram showing the transport of vesicles containing NPs within the cellular microtubule network. MTs are long tubulin polymers and are often anchored at the centrosome. Their plus ends extend toward the cell periphery, whereas their minus ends are located closer to the cell centre and are often anchored at the centrosome. Inset figure: Vesicular transport along MTs is supported by the motor proteins, dynein and kinesin. NP transport along actin filaments is supported by motor proteins closer to the cell periphery. (**b**) Representation of a single GNP functionalized with PEG and RGD peptides. (**c**) Snapshot of a live cell showing vesicles containing NPs (marked in red; left most), MTs (marked in green; middle), and the merged image (right most). Scale bar is 20 µm.

As illustrated in Figure 1a, MTs are long tubulin polymers that are polarized such that their plus ends extend toward the cell periphery and their minus ends exist closer to the cell centre and are often anchored at the centrosome, near the nucleus. MTs provide long tracks for transporting vesicles and organelles within the cell interior, while actin filaments near the cell periphery provide support for short distance transport [25,26]. Bidirectional transport along MTs is supported by two motor proteins, kinesin and dynein (see inset Figure 1a). Kinesins move cargo toward the (+) end of MTs, whereas dyneins transport cargo toward the (−) end. The myosin motor protein moves cargo along actin filaments closer to the cell periphery (see inset Figure 1a). After endocytosis, NPs trapped in vesicles are transported back and forth along MTs for processing and removal from the cell. The arrangement of the MT network, centrosome, and vesicles containing NPs is given in Figure 1a. A snapshot of NPs in vesicles attached to MTs in a live cell shows a strong presence of NPs closer to the centrosome, the primary microtubule organizing centre.

NP transport is heavily dependent on the MT network. Hence, the MT network is a good target for modulating NP dynamics to improve therapeutics [27,28]. If we were to successfully integrate both DTX and NPs into any NP-driven therapeutic approach, the following questions need to be answered:

(a) Does NP size matter?
(b) Will DTX concentration affect NP uptake, transport, and retention?
(c) Does the relative timing of DTX and NP inoculation matter?

To answer the above questions, we chose GNPs as our model NP system due to their biocompatibility and ease of tailoring size and surface functionalization [14,17,18,19]. Evidence of their promise in therapeutic and imaging applications has been mentioned previously [28,29].

## 2. Materials and Methods

### 2.1. Cell Culture

MDA-MB-231 and HeLa cells were bought from ATCC. All cells were maintained in high-glucose DMEM (HyClone) supplemented with 10% FBS (Gibco) and 1% penicillin/streptomycin (Gibco).

### 2.2. GNP Preparation and Modification

Spherical GNPs were prepared via a modified citrate reduction method [30]. First, 300 μL of 1% chloroauric acid (HAuCl_4_·3H_2_O) (Sigma-Aldrich, Oakville, ON, Canada) was added to 30 mL of double–distilled water and brought to boil on a hot plate while stirring. The amount of the reducing agent added varied depending on the size of the NPs.

For example, through optimization studies performed it was determined that the addition of 1 mL and 300 μL of 1% sodium citrate tribasic dehydrate (HOC (COONa)(CH_2_COONa)_2_·2H_2_O) (Sigma-Aldrich) synthesized 15 nm and 50 nm GNPs, respectively. After the color of the solution changed from dark blue to bright red, the solution was left to boil for another ten minutes while being stirred. Finally, the GNP solution was brought to room temperature while being stirred.

### 2.3. Nanoparticle Surface Modification

Polyethylene glycol (PEG) is added as a surface coating to GNPs to prolong blood circulation in vivo. [31]. In order to evade uptake by macrophage cells of the immune system, a minimum density of 1 PEG per nm^2^ is required on the GNP surface [32]. Absence of nonspecific protein adsorption in blood results in a prolonged blood circulation, which increases the chances for GNPs to accumulate within the tumour using its leaky vasculature. PEG that was 2000 Da molecular weight was used to coat the GNP surface because it is closer to the molecular weight of the other molecule coating the surface, the peptide used for improved uptake of NPs RGD (1760 Da; sequence: NH2-Cys-Lys-Lys-Lys-Lys-Lys-Lys-Gly-Gly-**Arg-Gly-Asp**-Met-Phe-Gly-COOH). A 0.01mg/mL PEG solution was prepared with thiol-terminated PEG methyl ether. The PEG solution was added to the GNP solution to achieve a density of 1 PEG per nm^2^. For 15 nm and 50 nm GNP, 706 and 6648 PEG were added per NP, respectively [33]. Characterization of NPs is given in Figure S2.

### 2.4. Docetaxel (DTX) and GNP Inoculation

6-well dishes were plated with 300k cells/well (MDA-MB-231) or 400k cells/well (HeLa). The next day, wells were inoculated with DTX (diluted from DMSO in PBS and media, DMSO concentration 0.04% *v*/*v*) to a final concentration of 10 and 50 nM. Control wells were inoculated with DMSO carrier diluted via same method. All wells were inoculated with either 15 or 50 nm GNPs diluted in media to a final concentration of 0.2 nM. Unless otherwise specified, inoculation with DTX and GNPs occurred concurrently, and exposure was carried out for 24 h at 37 °C.

### 2.5. Cytotoxicity Assay

The cells were seeded in black-walled 96-well plates (10000 cells/well, 50 µL media). Docetaxel was diluted from a stock solution of 0.10 mg/mL in DMSO (120 µM) serially in DMSO. Each solution was further diluted 7.2:100 into PBS and then 1:8 into media. In total, 40 µL was added to each well to a total volume of 90 µL. The plates were incubated for a further 24, 48, or 72 h. Thirty minutes before the end of each plate’s incubation period, 10 µL of resazurin dye (PrestoBlue, Thermo-Fisher) was added. The plate was incubated for 30 min in a Cytation plate reader, then fluorescence was measured using filters at Ex 530/25, Em 590/35 nm. Viable cells reduce the resazurin compound, and the fluorescence of the product correlates linearly to the number of viable cells. The cytotoxicity response curve was fit using the Growth Rate Inhibition metric, which modifies the standard IC metric to account for the effects of slow division times comparable to the length of the assay [34].

### 2.6. GNP Uptake and Retention Dynamics

6-well dishes were plated with 300k cells/well. The next day, wells were inoculated with DTX (diluted from DMSO in phosphate buffered saline (PBS; HyClone) and media, DMSO concentration 0.04% *v*/*v*) to a final concentration of 10 nM or 50 nM. Control wells were inoculated with DMSO carrier diluted via same method. All wells were inoculated with 15 nm GNP-RGD diluted in media to a final concentration of 0.2 nM.

All wells received GNP-RGD inoculation at the same time. One set of wells (6 h) received DTX 6 h prior to GNP-RGD inoculation, while the other set of wells (0 h) and the control wells (CTL) received their inoculations (DTX or DMSO carrier, respectively) concurrently with the GNP-RGDs.

After incubation times of 1, 4, 8, or 24 h, the media was aspirated. Each dish was gently washed 3 times with PBS, then cells were detached with 1 mL of Trypsin. Cell concentrations were counted using an automatic cell counter (Beckman Coulter, Biotek) with a 100 uL sample of the suspension. The remaining solutions were processed by aqua regia in a heated mineral oil bath for 30 min and diluted into 5 mL total volume with deionized water. Gold concentration was measured using Inductively Coupled Plasma–Mass Spectroscopy (ICP-MS) (Optima 7300 DV, Perkin Elmer, Woodridge, Canada).

### 2.7. Quantification of Uptake in Cells

The accumulation of GNPs in cells was analyzed using Inductively Coupled Plasma Mass Spectrometry (ICP-MS). The ICP source converts the atoms of the elements in the sample into ions, which are separated and detected using a mass spectrometer as mentioned in the previous section.

Following 20-h incubation with GNPs, cells were washed three times with PBS, to ensure any remaining gold in the media was removed. The cells were then harvested into a single cell suspension using Trypsin 0.25% (HyClone), and the concentration of cells was determined using Beckman Coulter Z2 Particle Counter and Size Analyzer. For the ICP-MS analysis process, the cells needed to be digested and this was done using Aqua Regia in 3:1 ratio HCl:HNO_3_, in an oil bath at 200 °C. The samples were diluted and concentrations of gold (Au) atoms were measured in ppb with the Thermo X-Series II (X7) quadrupole ICP-MS instrument. A calibration curve was created using standards prior to sample measurements.

The following equations were used to calculate the number of GNPs of each sample from the concentration of Au atoms measured from ICP-MS:

(1) Number of Au atoms per GNP (U)=Number of Atoms per unit cell (∗)×Volume of GNP (sphere) Volume of unit cell =4×4π (D2)33a3=23π(Da)3

where D = core diameter of GNP, a = length of a unit cell = 4.08 Å = 0.408 nm.

* Gold nanoparticles synthesized through salt reduction methods assemble into Face-Centered Cubic (FCC) structures and FCC lattices contain 4 atoms per unit cell (a unit cell refers to the smallest repeating structure of any solid used to simplify the crystalline patterns solids arrange themselves to a lattice) [35].

(2) $$\begin{array}{l} \mathrm{Number}\;\mathrm{of}\;\mathrm{GNPs}\;\mathrm{for}\;\mathrm{each}\;\mathrm{sample}\\ \;=\;\text{conc}.\;\text{measured}\;\text{from}\;\text{ICP}\;\text{MS}\;\left[\frac{g}{L}\right]\times \text{volume}\;\text{measured}\;\left(\text{L}\right)\\ \;\times \frac{1}{\text{molar}\;\text{weight}\;\text{of}\;\text{Au}}\;\left[\frac{\text{mol}}{\text{g}}\right]\times {\text{Avogadr}}^{’}\text{s}\;\text{number}\;\left[\frac{\text{Au}\;\text{atoms}}{\text{mol}}\right]\\ \;\times \frac{1}{\text{U}}\;\left[\frac{\text{GNP}}{\text{Au}\;\text{atoms}}\right] \end{array}$$

The number of GNPs per cell is then determined by dividing the number of GNPs by the total number of cells for that sample. This calculation assumes a homogenous distribution of GNPs in the cell population [36].

### 2.8. Confocal Imaging

A laser scanning confocal microscope (NIKON Eclipse TE2000-U) was used for live cell imaging. For optical imaging, 50% of the PEG on the GNP surface was replaced with PEG-Cy5 complex (excitation 633 nm, emission filter 650 nm LP). For MT imaging, α-tubulin was labelled with a viral transfection stain (CellLight Tubulin-GFP, BacMam 2.0, obtained from Thermo-Fisher). Since most MT stains are taxane-based, we chose this particular viral stain to avoid competing effects with DTX binding sites.

Glass bottom coverslip dishes (MatTek, Ashland, MA, USA) were used to facilitate live cell imaging. The media used was FluoroBrite DMEM (Gibco) supplemented with 10% FBS (Gibco) and 1% penicillin/streptomycin (Gibco). Cells were first incubated with the viral stain for 24 h before adding DTX and optically labelled GNPs. Image processing was performed using ImageJ. It can be seen in images that not all the cells are labelled with the MT stain. This is due to the lower transfection efficiency of the viral stain used for staining MTs.

### 2.9. Measuring DNA Damage

We probed the DNA damage repair protein 53BP1 for mapping the DNA damage. A primary antibody and an optically labeled secondary antibody were used for the assay. The cells were grown on cover slips for imaging experiments. Once they were adhered to glass coverslips, fresh media with 0 and 0.2 nM GNPs was added and the cells were incubated for a 24 h time period. Following the incubation, the cells were washed with PBS three times and fixed with 4% PFA for 5 min at room temperature. The cells were then washed with PBS three times and treated with 2% BSA/0.1% Triton-X in PBS for 20 min. The 53BP1primary antibody was diluted 1:200 in 0.5% BSA/0.1% Triton-X/PBS, while the two secondary antibodies were diluted 1:500 in 0.5% BSA/0.1% Triton-X/PBS. The coverslips were first incubated with the primary antibody, followed by washing with PBS three times. The cells were rinsed twice with 0.5% BSA/0.175% Tween-20/PBS for 5-min time durations and incubated with secondary antibody in the dark for 30 min. Following the incubation, the cells were rinsed in PBS, dried, and mounted to glass coverslips with Prolong Glass for imaging.

### 2.10. Measuring Cell Survival Fraction

We used a clonogenic assay for measuring the cell survival fraction. After the treatments, the cells were trypsinized and diluted to form single-cell suspensions. Cell concentrations were determined by counting using a hemocytometer. The required volumes of cell suspension solution were calculated for the control and treatment samples. The calculated volume of cell suspension for each condition was seeded in 60 mm tissue culture dishes in triplicate. In total, 200 cells were plated for control conditions and 1000 to 5000 cells were plated to be treated with DTX. The cells were left in the 37 °C humidified incubator with 5% CO_2_ for 14 days for colonies to grow. Once colonies were formed, the dishes were stained and fixed with 0.1% of methylene blue (BioShop) in 70% ethyl alcohol for 1 h. The stained dishes were rinsed in lukewarm water and left to air-dry overnight. The air-dried control dishes were then counted. Colonies were defined as structures containing >50 cells. The plating efficiency (PE) was then obtained with the following equation:

(3) $$\mathrm{PE}=\frac{\mathrm{Number}\;\mathrm{of}\;\mathrm{colonies}\;\mathrm{counted}}{\mathrm{Number}\;\mathrm{of}\;\mathrm{cells}\;\mathrm{plated}}$$

The colonies of treatment samples were also counted and the survival fraction (SF) was obtained with the following equation:

(4) $$\mathrm{SF}=\frac{\mathrm{Number}\;\mathrm{of}\;\mathrm{colonies}\;\mathrm{counted}}{\mathrm{Number}\;\mathrm{of}\;\mathrm{cells}\;\mathrm{plated}\;\times \mathrm{PE}}$$

## 3. Results and Discussion

### 3.1. Effect of NP Size and Docetaxel Concentration on Intracellular Uptake of NPs

**Figure 2:**
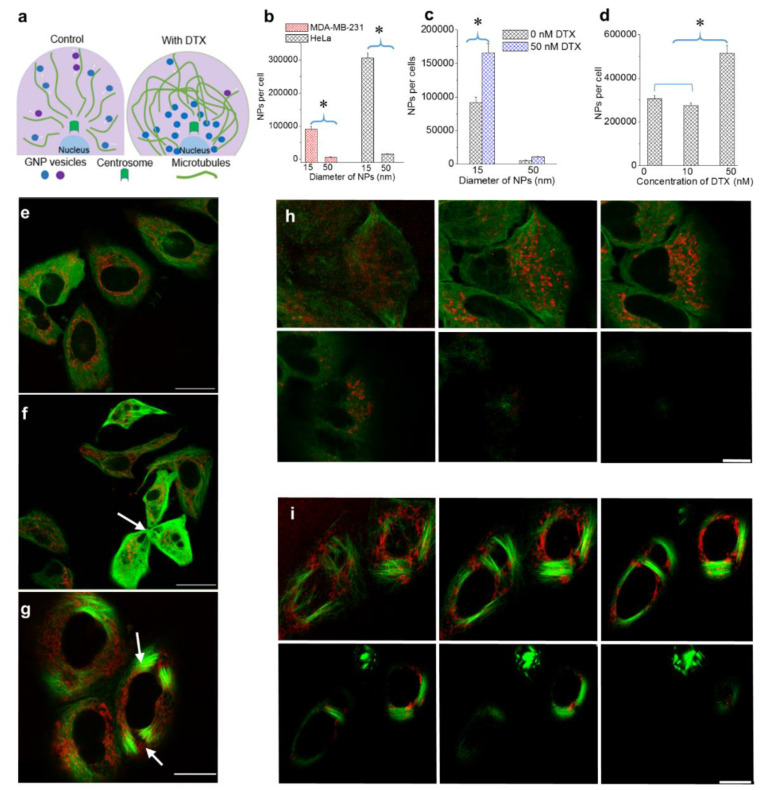
The effect of NP size and DTX concentration on intracellular NP accumulation. (**a**) Schematic diagram highlighting differences in the MT network and NP distribution in the absence and presence of DTX. (**b**) Size-dependent uptake of NPs in MDA-MB-231 and HeLa cells. (**c**) Size-dependent uptake of NPs in the presence of a 50 nm DTX concentration in MDA-MB-231 cells. (**d**) DTX concentration dependent NP uptake in MDA-MB-231 and HeLa cells. (**e**–**g**) Images showing NPs and the MT network in cells treated with 0, 10, and 50 nM of DTX, respectively (**f**): arrow indicates cell fragmentation during division; g: arrows indicate bundling of MTs). (**h**–**i**) Z-stack showing the distribution of NPs and the MT network in a group of cells treated with 0 and 50 nM DTX, respectively. Vesicles containing GNPs and MTs are marked in red and green, respectively. Scale bar is 20 µm. Error bars are standard deviations from 3 replicate measurements. * Represents a statistically significant difference (Welch’s unequal variance *t*-test, *p* < 0.05).

Most NP systems enter the cell via endocytosis, become trapped in endosomal vesicles, fuse with lysosomes for processing, and finally are removed from the cell through exocytosis [24]. As explained in schematic Figure 2a, vesicular transport of NPs is expected to be significantly affected by the presence of the MT stabilizing drug, DTX. It is also known that NPs’ size plays a significant role in their regular endo-exo process [24]. However, it not yet known how NP size and MT stabilization agent concentration affect NP behaviour within cells. We chose NPs of diameters 15 and 50 nm to investigate the size-dependent uptake. Nanoparticles were functionalized with both polyethylene glycol (PEG) and a peptide containing the integrin binding domain RGD (referred to as an RGD peptide) as illustrated in Figure 1b. The PEG and RGD peptide were 2 and 1.7 kDa in size, respectively. Characterization data for NP complexes is provided in Figure S1.

Our first goal was to map the size-dependent uptake of NPs within a regular MT network as shown in Figure 1c and Figure 2a (left side). We used a triple negative breast cancer cell line, MDA-MB-231, and a cervical cancer cell line, HeLa, for this study. The details of the approach used and quantification of the NPs per cell are given in the methods section. There was an over 15-fold increase in the uptake of smaller NPs as compared to larger ones in both cancer cell lines (see Figure 2a,b). This size-dependent effect is consistent with previously published work and the outcome is a result of variation in receptor-ligand interaction as a function of surface curvature (or size) of NPs [31]. The higher surface curvature of smaller NPs enabled efficient interaction between the targeting ligand, RGD, and cell surface integrins. In contrast, the targeting ligand could be hidden by PEG on a larger NP surface due to its lower surface curvature, thus overall reducing receptor-ligand interaction.

Our next goal was to study the changes in NP transport behaviour as a function of NP size and DTX concentration. We first looked at the effect of NP size within a stabilized MT network (Figure 2a (right side)). In order to investigate the size-dependent uptake, we used a DTX concentration of 50 nM. Based on our growth delay experiments, this concentration was determined to be sufficient to cause maximal disturbance to the MT network with limited toxicity. Furthermore, such drug concentrations can be achieved in vivo as well [33,37,38]. Size-dependent NP cellular uptake was studied using 15 and 50 nm diameter GNPs. The NP concentration was 0.2 nM. NP accumulation was conducted via simultaneous incubation of NPs and DTX over a 24 h period. According to Figure 2c, the presence of DTX improved the accumulation of both 15 and 50 nm NPs significantly. Smaller NPs still maintained a significantly higher uptake compared to larger ones. These results can be explained as follows:

(a) Following treatment with DTX, mitosis is arrested during metaphase. The prolonged time in M phase enables greater accumulation of NPs within cells. This led to the increase in uptake of NPs of both sizes.
(b) The presence of DTX did not significantly affect the endocytosis process since it is a process largely governed by the actin cytoskeleton closer to the cell periphery [39]. The cells’ ability to maintain efficient endocytosis enabled significantly higher accumulation of smaller NPs in DTX-treated cells (seen in Figure 2c).

In order to investigate the concentration dependence of DTX on intracellular NP behaviour, we chose to use GNPs of a 15 nm diameter (due to their favourable intracellular accumulation). The chosen DTX concentrations were 0, 10, and 50 nM. According to Figure 2d, we observed reduced GNP uptake when the cells were treated with 10 nM DTX, as compared to the 0 and 50 nM DTX conditions in HeLa cells (see Supplementary Materials S1 for results corresponding to MDA-MB-231). The reduced accumulation of NPs in cells treated with 10 nM DTX, as compared to 0 nM DTX, could be due to the fragmented division seen in cells treated with 10 nM DTX (Figure 2f) [40]. The greater accumulation of NPs in cells treated with 50 nM DTX could be due to the cell cycle arrest in G2/M phase [20,40,41]. We also examined the NP accumulation when cells were treated with 1 nM DTX and observed an outcome much closer to the control and 10 nM conditions, as expected (see Supplementary Materials S2).

The distribution of NPs in cells treated with 0, 10, and 50 nM DTX is shown in Figure 2e–g, respectively. The images display the NP distribution and MT network across a single imaging plane. The variation of cell morphology, NP distribution, and MT network across many planes of a cell population treated with 0 and 50 nM DTX is given in Figure 2h,i, respectively. In control cells (Figure 2e,h), NPs were distributed throughout the MT network. However, more NPs were observed to have clustered around the centrosome in a manner similar to what was observed in Figure 1c (similar behaviour was seen in MDA-MB-231 cells; see Figures S3–S10). Figure 2f shows a possible cell fragmentation of a parent cell to three daughter cells with the treatment of 10 nM DTX (marked by an arrow). The bundling of MTs and the resulting change in cell shape were clearly observed in cells treated with 50 nM DTX (Figure 2g,i; arrows indicate bundling of MTs; similar behaviour was seen in MDA-MB-231 cell line (see Supplementary Materials S3–S5)). GNPs were driven towards the nucleus or became trapped in areas where there were no MTs. Within this population of cells, we investigated the NP distribution in cells which had either divided or were in early stages of the cell cycle. As explained in the next section, we were also able to capture the NP distribution during mitosis.

### 3.2. Distribution of Nanoparticles During Cell Division (Mitosis)

**Figure 3:**
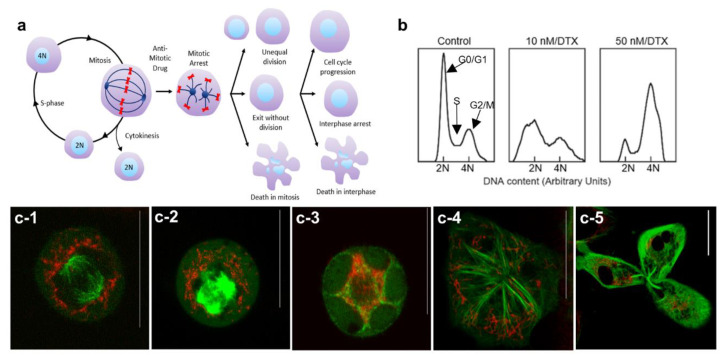
Distribution of NPs during cell division in the presence of DTX. (**a**) Schematic diagram illustrating the different outcomes of cell division in the presence of the antimitotic drug, DTX. (**b**) Cell cycle analysis for control cells and cells treated with 10 and 50 nM concentrations of DTX. (**c**) Distribution of NPs in a control cell (**c-1**), cells treated with 50 nM DTX (**c-2**–**c-4**) and in a cell treated with 10 nM DTX (**c-5**). Vesicles containing GNPs and MTs are marked in red and green, respectively. Scale bars are 20 µm.

The cell cycle can be divided into four major phases; G1, S, G2, and M (see Supplementary Materials S1). The genetic information is duplicated during the synthesis (S) phase and the cell divides into two daughter cells during mitosis (M). MT dynamics play a critical role during cell division as illustrated in Figure 3a. Hindering MT dynamics with antimitotic drugs, such as DTX, could lead to many different outcomes including exit from mitosis without division, unequal division, and even cell death during mitosis. Cell fate depends on the antimitotic drug used and on its concentration, but not on the duration of mitotic arrest [42]. Based on our cell cycle analysis data (Figure 3b) and images (Figure 3c), the majority of cells treated with 50 nM DTX were halted in G2/M phase, while some cells treated with 10 nM DTX were still able to complete mitosis (see Figure 3b).

**Figure 4:**
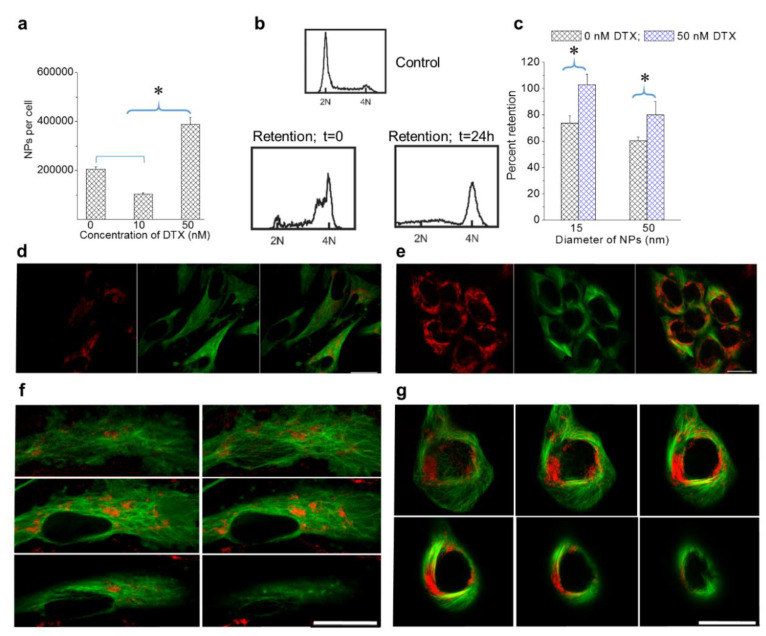
Retention of NPs in cells treated with DTX. (**a**) Retention of NPs in DTX-treated cells after 24 h. (**b**) Cell cycle analysis for control HeLa cells and cells treated with 50 nM DTX at the beginning and end of the retention. (**c**) NP size-dependent retention in MDA-MB-231 cells treated with 50 nM DTX. (**d**,**e**) Images corresponding to control cells and cells treated with 50 nM DTX after 24 h, respectively. (**f**,**g**) Z-stack showing the distribution of NPs in different planes following the retention process in a control cell and a cell treated with 50 nM DTX, respectively. Vesicles containing GNPs and MTs are marked in red and green, respectively. Scale bar is 20 µm. Error bars are standard deviations from 3 replicate measurements. * Represents a statistically significant difference (Welch’s unequal variance *t*-test, *p* < 0.05).

**Figure 5:**
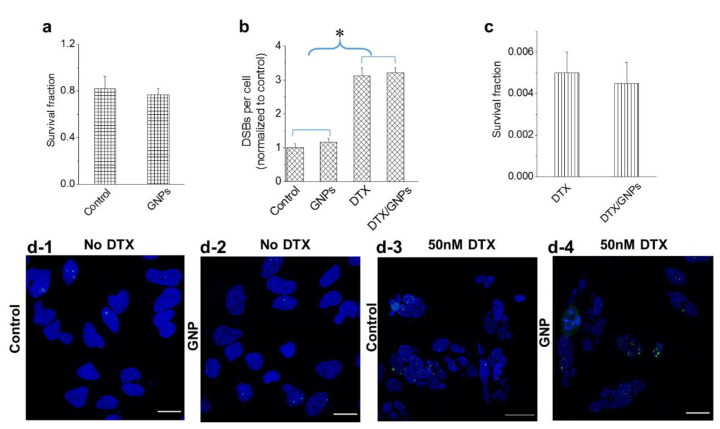
Action of DTX in the presence of GNPs. (**a**,**b**) The biocompatibility of GNPs was verified by measuring the cell survival fraction and DNA damage, respectively. (**b**,**c**) The action of the drug in the presence of GNPs was verified by measuring the DNA damage and cell survival fraction, respectively. (**d**) Mapping of DNA double strand breaks (DSBs) in control cells and cell treated with 50 nM DTX. DNA DSBs and nuclei are marked in green and blue, respectively. Scale bar is 20 µm. Error bars are standard deviations from 3 replicate measurements. * Represents a statistically significant difference (Welch’s unequal variance *t*-test, *p* < 0.05).

We were able to capture the arrangement of microtubules and the distribution of NPs during mitosis in control cells and cells treated with DTX (Figure 3c). During a normal mitosis process, the interphase cytoskeletal MT array is disassembled, and a bipolar spindle is assembled (see Figure 3a,c-1). Spindle MTs attach to chromosomes to support the alignment and subsequent segregation of chromosomes to form two daughter cells and NPs became quite evenly redistributed around the exterior of the bipolar spindle to facilitate their inclusion in the daughter cells (Figure 3c-1). NPs were not observed within the mitotic spindle apparatus. In cells treated with 50 nM DTX, the formation of asters was observed instead of the bipolar spindle (Figure 3c-2). We also observed multinucleation as the nuclear envelope reformed around the multiple asters (see Figure 3: c-3,c-4). NP distribution was uneven in DTX-treated dividing cells. A DTX concentration of 10 nM could still allow mitosis to proceed but sometimes led to uneven cell division. An example of this is shown in Figure 3c-5 where a cell was dividing into three daughter nuclei with uneven distribution of NPs among them. 

### 3.3. The Effect of the Docetaxel Concentration on the Intracellular Retention of NPs

The retention of NPs within cells is essential to capturing the full therapeutic benefit of many NP-driven treatment strategies. As discussed in the introduction, the presence of DTX could alter the MT dynamics and thus induce changes to NP processing and transport [25,26]. More specifically, DTX seems to induce NP transport towards the cell periphery for removal. This is expected to cause an increase in NP retention as a result of reduced exocytosis. In order to test our hypothesis, we incubated cells with NPs and DTX (0, 10, and 50 nM DTX) over a 24-h time period, washed them with phosphate buffered saline three times to remove NPs that were not internalized, and then incubated them with fresh media for another 24-h period to evaluate retention. The control cells (0 nM DTX treated) retained approximately 40% of their original NP content, which was consistent with previous studies (based on Figure 2d and Figure 4a; see Supplementary Materials S5 for data corresponding to MDA-MB-231) [43]. In the condition where cells were treated with 10 nM DTX, a lower number of NPs were retained per cell as compared to cells treated with 0 and 50 nM of DTX (Figure 4a). As discussed in the previous section, this was most likely due to the fragmentation of some cells lowering the number of NPs per cell in an average population. However, cells treated with 50 nM DTX were able to retain over 90% of their NP content. According to Figure 4b, most of the cells were in G2/M phase even at the end of retention experiment. Hence, the significantly higher retention could be mainly due to the halt in cell division and the cells’ inability to remove NPs from themselves due to the defects in their MT structure. Cell cycle data in Figure 4b further confirms that the majority of cells remained in G2/M phase even following 24-h exposure to DTX.

We also investigated the size dependence of NP retention at 0 and 50 nM DTX (Figure 4c). Smaller NPs had higher retention as compared to larger NPs. Most importantly, cells treated with DTX retained over 90% of their NPs. This is quite promising for NP-based applications, as DTX is usually administered weekly to patients. The promising results of this study open up the possibility of fewer NP injections for a given treatment due to the fact that DTX enables greater retention of NPs within cells. This could be particularly useful in GNP-mediated fractionated radiotherapy.

Image panels in Figure 4d,e show the retention of NPs in cells treated with 0 and 50 nM DTX. Even after 24 h, the strong NP presence in cells formerly treated with 50 nM DTX can be seen based on the images in Figure 4e. Furthermore, the shape of the cells and the distribution of NPs were quite similar to the images seen in Figure 2g. The distribution of NPs across many planes of a control cell and a cell initially treated with DTX are given in Figure 4f,g respectively. The significant enhancement in NP uptake and retention following treatment with 50 nM DTX, as compared to 10 nM DTX, suggests that the use of 50 nM DTX will have more optimal therapeutic outcomes.

### 3.4. Effect of GNPs on the Action of the Drug Docetaxel

We also investigated whether the stronger GNP presence in cells treated with 50 nM DTX could influence the action of the drug using both a clonogenic assay and a DNA double strand breaks (DSBs) assay (Figure 5). The biocompatibility of GNPs at a 0.2 nM concentration was evident based on the insignificant differences in cell survival (Figure 5a) and the extent of DSBs (Figure 5b) in treated cells as compared to the control. We probed the extent of DNA DSBs using an optically labeled antibody against the repair protein, 53BP1 (see methods section). Following treatment with DTX, there was an increase in DNA DSBs as compared to the control (Figure 5b). Although DNA damage occurs constantly in cancer cells, the majority of it is repaired by DNA damage repair proteins, such as 53BP1, trafficked on microtubules [44]. When the cells were treated with DTX, this transport of these repair proteins was hindered resulting in an increase in DNA DSBs. It is also important to notice that the action of DTX was not affected by the presence of GNPs (Figure 5b,c). Qualitative images in Figure 5d solidify the quantitative data presented in Figure 5b. The foci (marked in green) formed within nuclei (marked in blue) are an indication of the extent of DNA DSBs.

### 3.5. The Relative Timing of DTX and GNP Inoculation 

**Figure 6:**
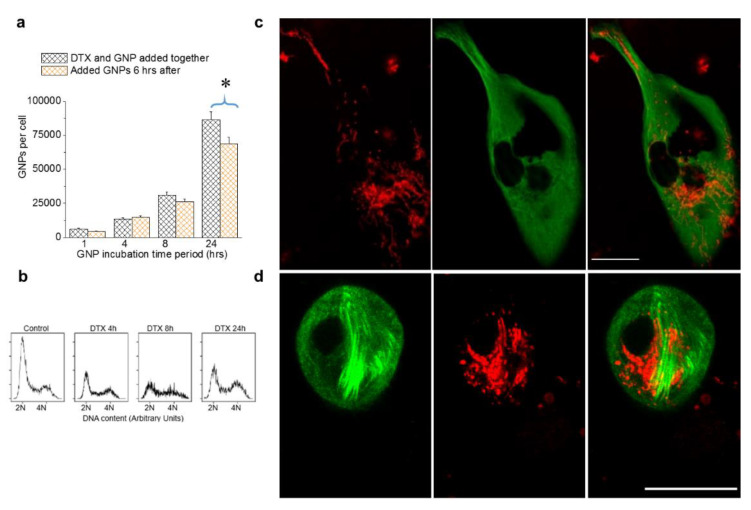
The relative timing of DTX and GNP inoculation in MDA-MB-231 cells treated with 0 and 50 nM DTX. (**a**) Variation in intracellular GNP accumulation with simultaneous addition of GNP and DTX vs. GNPs added 6 hrs after the addition of DTX. (**b**) Cell cycle analysis for control cells and cells treated with 50 nM DTX after 4, 8, and 24 h. (**c**,**d**) Images of individual cells treated with 0 and 50 nM DTX, respectively. Vesicles containing GNPs and MTs are marked in red and green, respectively. Scale bar is 20 µm. Error bars are standard deviations from 3 replicate measurements. * Represents a statistically significant difference (Welch’s unequal variance *t*-test, *p* < 0.05).

DTX is a widely used drug to treat breast cancer. In our experiment, we employed the simultaneous incubation of NPs and DTX in a triple negative breast cancer cell line, MDA-MB-231. At 50 nM DTX, we observed a significant increase in NP uptake and retention without causing any additional toxic effects (Figure 4 and Figure 5). Based on these results, we aimed to investigate what would happen if we introduced GNPs after administering the drug vs. the simultaneous administration of the drug and GNPs. It was observed that there were minimal differences in NP uptake between the two treatment approaches up to 8 h. After 24 h, there was a ~20% increase in NP uptake with simultaneous treatment with GNPs and DTX vs. administering GNPs 6 h after introducing the drug (Figure 6a). We also probed the dynamics of cell cycle progression at 50 nM DTX, and it was clear that DTX can arrest most of the cells in mitosis within 4 h of incubation (Figure 6b). This suggests that part of the cell population was already undergoing mitosis when GNPs were introduced. Clathrin-mediated endocytosis is halted during mitosis. Hence, while the mitotic arrest from DTX prevents redistribution of GNPs into daughter cells, it also halts uptake until the cell undergoes mitotic catastrophe. By administering GNPs after DTX, the uptake potential of a significant population of cells is lost as they are already arrested. This could explain the lower uptake of NPs when GNPs were introduced after administering the drug. In previous figures, we presented images of HeLa cells to demonstrate the differences in NP distribution and MT network when cells were treated with DTX. Images corresponding to MDA-MB-231 were placed in the Supplementary Materials Section S3 and S4. The imaging data related to both cell lines were consistent. In order to complete the picture, we have placed images of MDA-MB-231 cells treated with 0 and 50 nM in Figure 6c,d.

## 4. Conclusions

Our study demonstrated how we can employ a widely used clinical drug, DTX, to enhance the accumulation and retention of NPs within cancer cells. This effect was DTX concentration dependent. A 50 nM concentration of DTX enabled maximization of the cellular NP dose while minimizing cellular toxicity. It is also critical to investigate the effect of NP size on uptake, since it is likely that both the NP size and the molecules used for NP functionalization could play a significant role in receptor-ligand integration. In this study, we showed that 15 nm NPs were more efficient in entering cells, as compared to 50 nm NPs. The combined use of 15 nm diameter GNPs and a 50 nM concentration of DTX has promise for improved outcomes in future therapeutics. It was also noted that GNPs did not influence the action of the drug. Although we used GNPs as our model system, this outcome could be applied to other NP systems as well. For example, there are over 15 approved drugs that could be classified as nanomedicines [2,3,45]. While concurrent delivery of free DTX and GNPs was used in this study, DTX could also be ligated directly to GNPs for combined delivery with fewer side effects. This has been done recently with a paclitaxel, a related drug [4]. Furthermore, clinical trials based on combinatorial treatment of radiotherapy and DTX are already underway to improve the therapeutic outcome while minimizing side effects through reducing individual doses [46,47,48,49,50,51,52]. The addition of GNPs to these protocols is possible considering their biocompatibility. These novel nanomedicine-based platforms would improve delivery of therapeutics while minimizing the side effects associated with current treatment options and thereby improve patient quality of life and response [53,54].

## Supplementary Materials

The following are available online at https://www.mdpi.com/2306-5354/7/2/56/s1, Figure S1: Changes to the phase cell population with the treatment of DTX. (a) Regular cell cycle: As a cell prepares for division it goes through three different phases: G1 is the gap between M and S phase, DNA replication occurs in S phase and G2 is when the cell prepares for mitosis. (b,c) Spread of the phases within a control cell population and one treated with 50 nM DTX, respectively; Figure S2. (a,b) TEM images of GNPs of diameter 15 and 50 nm, respectively. (c) UV-Visible peak wavelength and hydrodynamic diameter for as-made (GNP), GNP-PEG, and GNP-PEG-RGD. Scale bars are 50 nm. Figure S3. Nanoparticle uptake as a function of DTX concentration in both HeLa and MDA-MB-231. Figure S4. Dynamics of nanoparticle uptake as a function of DTX concentration. MDA-MB-231 was used as the cell line and nanoparticles of diameter 15 nm were used. DTX concentrations used were 0, 1, and 50 Nm; Figure S5. MT network and distribution of NPs in MDA-MB-231 cells treated with 0, 10, and 50 nM DTX. Scale bar is 20 µm. Vesicles containing GNPs and MT network are marked in red and green, respectively; Figure S6. MT network and distribution of NPs in MDA-MB-231 control cells (0 nM DTX) across many planes of a cell. Scale bar is 20 µm. Vesicles containing GNPs and MT network are marked in red and green, respectively; Figure S7. MT network and distribution of NPs across many planes of a cell treated with 50 nM DTX. Scale bar is 20 µm. Vesicles containing GNPs and MT network are marked in red and green, respectively; Figure S8. (a,b) Variation of nanoparticle distribution and cell morphology in Hela (top panel) and MDA-MB-231 (bottom panel) cells treated with 0 and 50 nM DTX, respectively. Scale bar is 20 µm. Vesicles containing GNPs and MT network are marked in red and green, respectively; Figure S9. (a–c) Variation of nanoparticle distribution and cell morphology in Hela cells treated with 0 (top panel), 10 (bottom left), and 50 (bottom right) nM DTX, respectively. Scale bar is 20 µm. Vesicles containing GNPs and MT network are marked in red and green, respectively; Figure S10. Retention of nanoparticles as a function of DTX concentration in both HeLa and MDA-MB-231.
