# Supplementary material for: Modulation of the Microtubule Network for Optimization of Nanoparticle Dynamics for the Advancement of Cancer Nanomedicine
Source: Bioengineering (Basel). 2020 Jun 14;7(2):56. doi: 10.3390/bioengineering7020056 (PMC7355834; doi:10.3390/bioengineering7020056)
Supplement: Supplementary file 1 [file bioengineering-07-00056-s001.pdf]

Modulation of the microtubule network for optimization of nanoparticle dynamics for the advancement of cancer nanomedicine

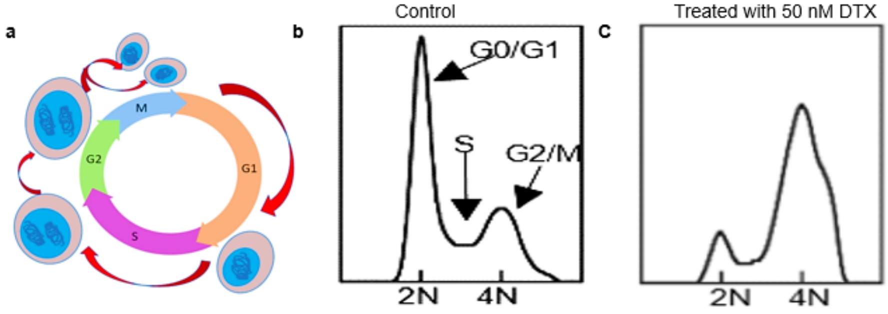

Figure S1. Changes to the phase cell population with the treatment of DTX. a) Regular cell cycle: As a cell prepares for division it goes through three different phases: G1 is the gap between M and S phase, DNA replication occurs in S phase and G2 is when the cell prepares for mitosis. b-c) Spread of the phases within a control cell population and one treated with 50 nM DTX, respectively.

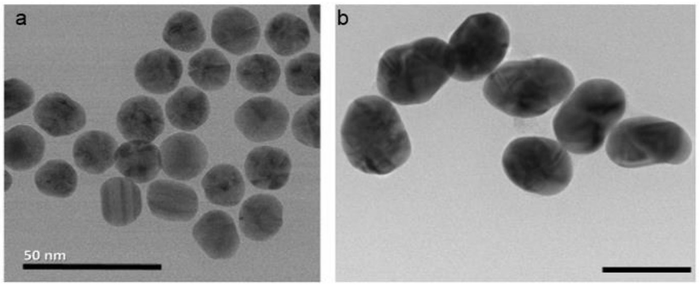

c

Nominal size of the NP (nm)	Peak absorption wavelength (nm); UV visible			Hydrodynamic diameter (nm)		
	As made	GNP-PEG	GNP-PEG-RGD	As made	GNP-PEG	GNP-PEG-RGD
15	518.80 ± 0.06	521± 0.08	529 ± 0.05	22.35 ± 3.55	33.15 ± 5.23	35.25 ± 6.45
50	532.30 ± 0.08	533 ± 0.07	533 ± 0.06	54.52 ± 5.32	55.45 ± 6.46	57.10 ± 6.64

Figure S2. a–b) TEM images of GNPs of diameter 15 and 50 nm, respectively. c) UV-Visible peak wavelength and hydrodynamic diameter for as-made (GNP), GNP-PEG, and GNP-PEG-RGD. Scale bars are 50 nm.

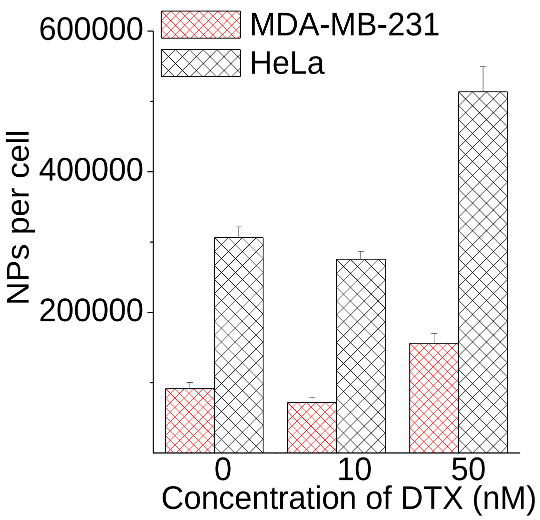

Figure 3. Nanoparticle uptake as a function of DTX concentration in both HeLa and MDA-MB-231.

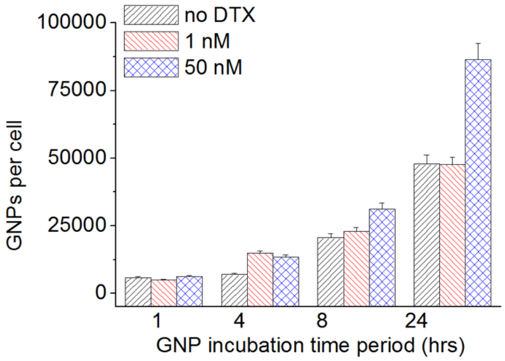

Figure S4. Dynamics of nanoparticle uptake as a function of DTX concentration. MDA-MB-231 was used as the cell line and nanoparticles of diameter 15 nm were used. DTX concentrations used were 0, 1, and 50 nM.

Most of the images presented in the paper were corresponding to HeLa cell. This section contains images for MDA-MB-231 cells under different concentrations of DTX.

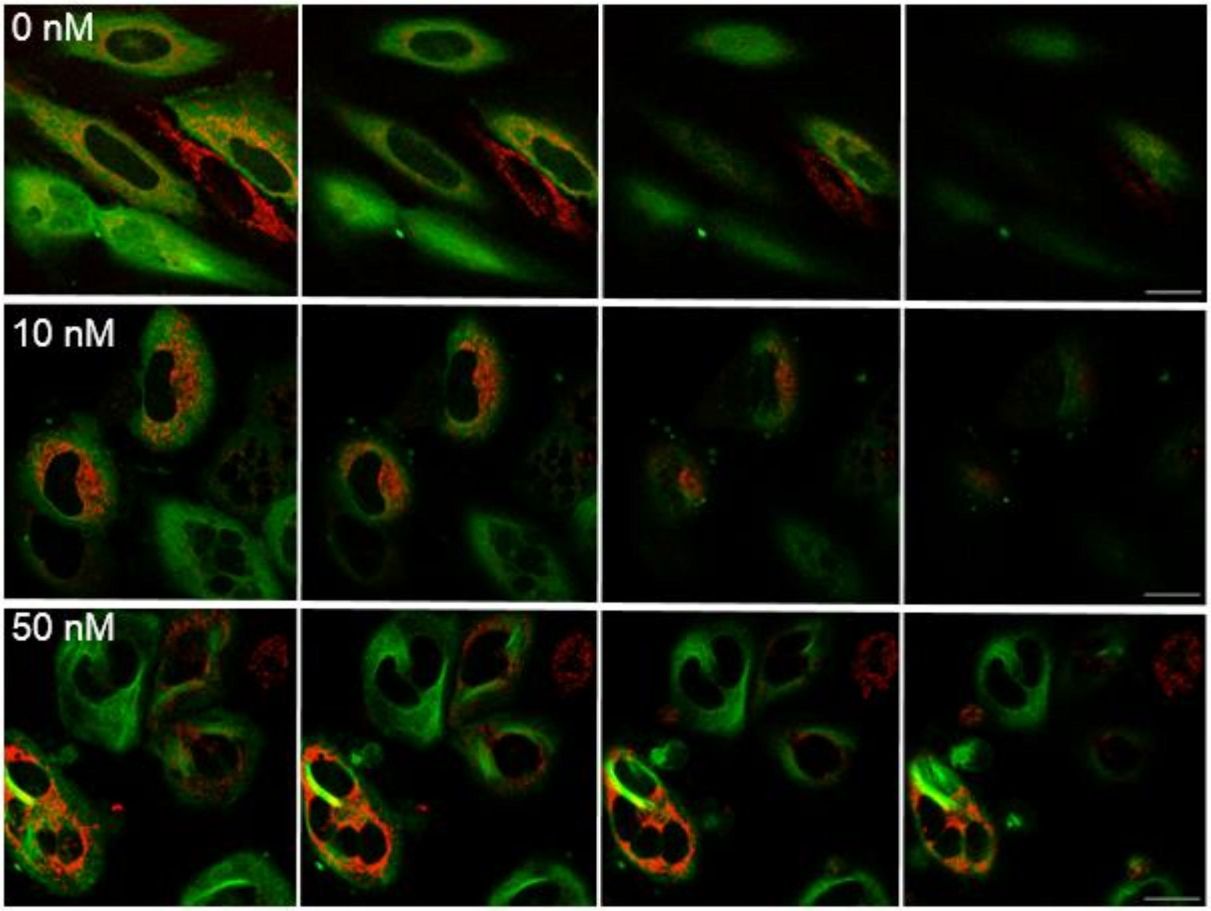

Figure S5. MT network and distribution of NPs in MDA-MB-231 cells treated with 0, 10, and 50 nM DTX. Scale bar is 20 μm. Vesicles containing GNPs and MT network are marked in red and green, respectively.

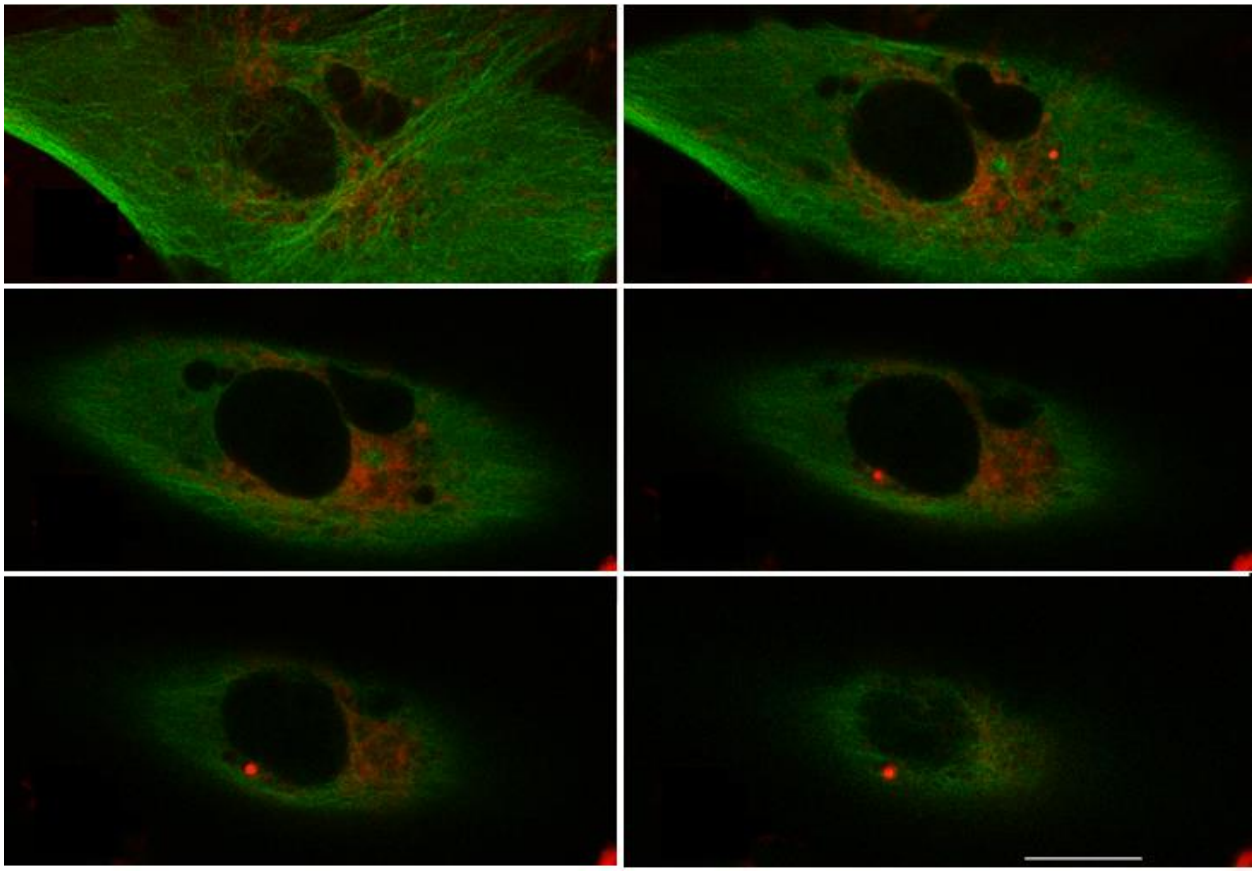

Figure S6. MT network and distribution of NPs in MDA-MB-231 control cells (0 nM DTX) across many planes of a cell. Scale bar is 20 μm. Vesicles containing GNPs and MT network are marked in red and green, respectively.

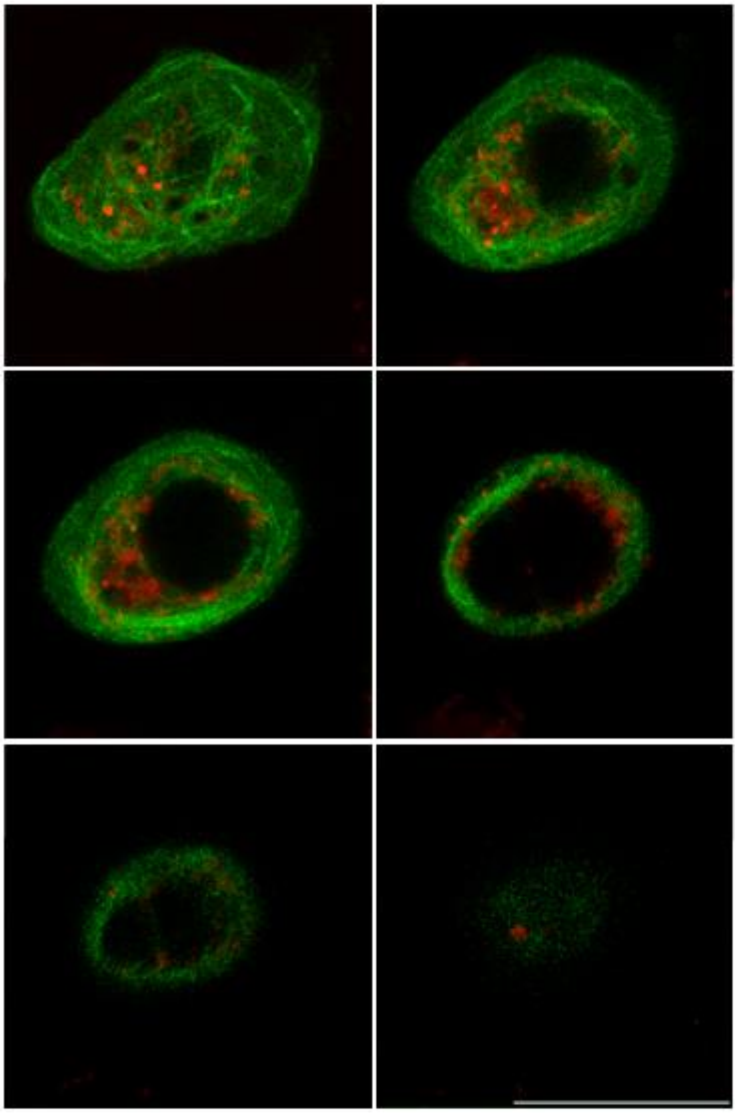

Figure S7. MT network and distribution of NPs across many planes of a cell treated with 50 nM DTX. Scale bar is 20 μm. Vesicles containing GNPs and MT network are marked in red and green, respectively.

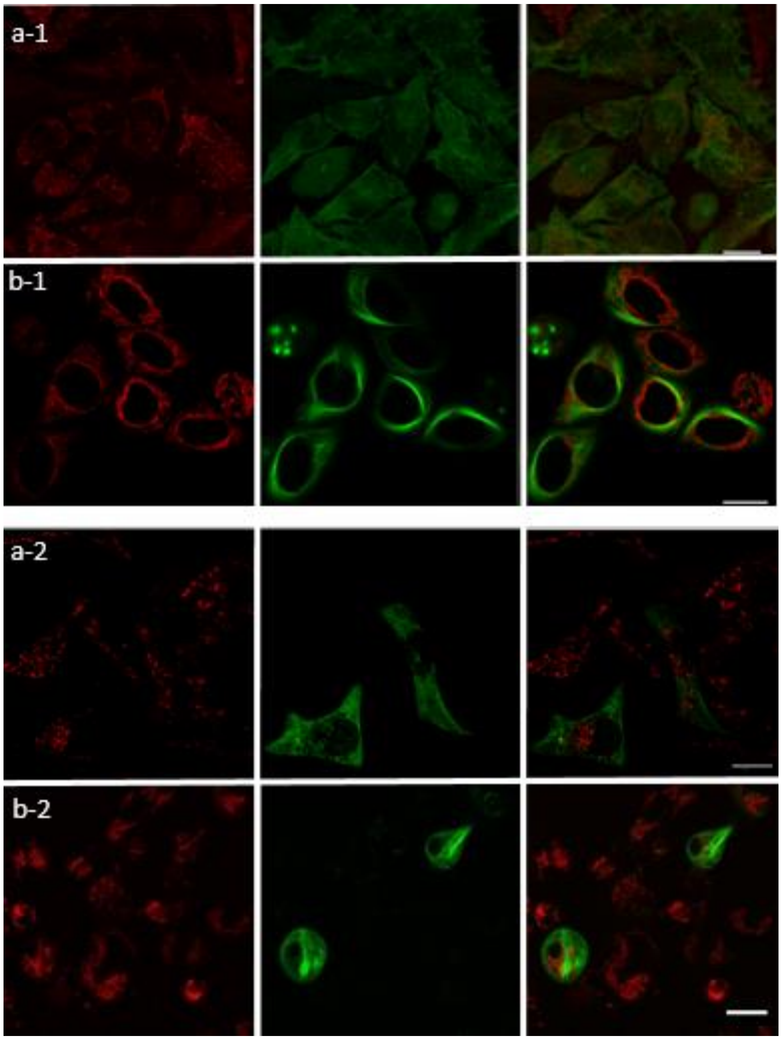

Figure S8. a-b) Variation of nanoparticle distribution and cell morphology in Hela (top panel) and MDA-MB-231(bottom panel) cells treated with 0 and 50 nM DTX, respectively. Scale bar is 20 μm. Vesicles containing GNPs and MT network are marked in red and green, respectively.

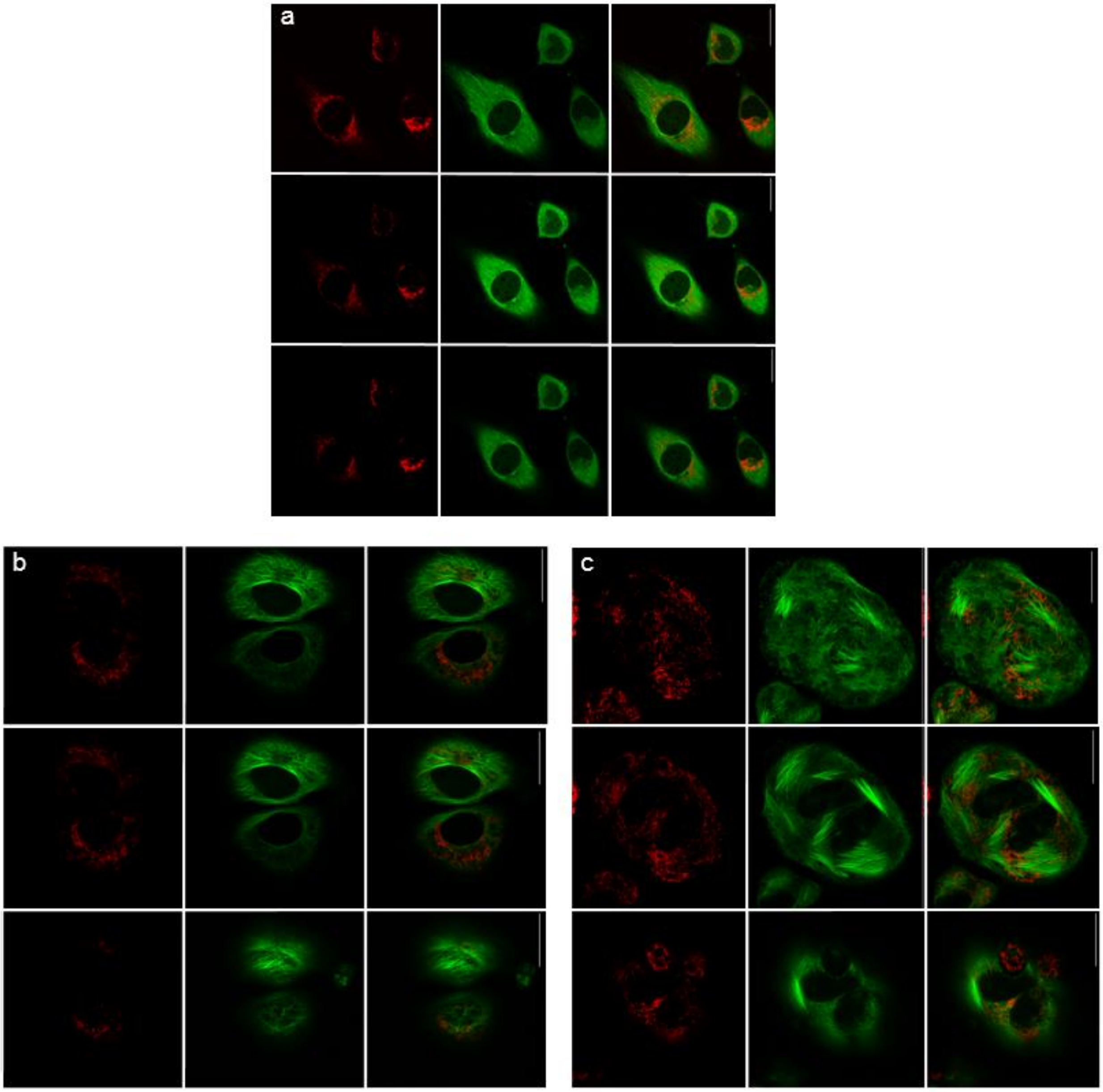

Figure S9. a-c) Variation of nanoparticle distribution and cell morphology in Hela cells treated with 0 (top panel), 10 (bottom left), and 50 (bottom right) nM DTX, respectively. Scale bar is 20 μm. Vesicles containing GNPs and MT network are marked in red and green, respectively.

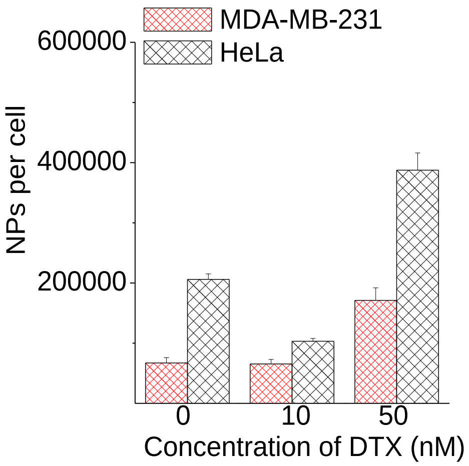

Figure S10. Retention of nanoparticles as a function of DTX concentration in both HeLa and MDA-MB-231.
